# Sebaceoma of the Eyelid Originating in a Meibomian Gland: A Rare Case and Literature Review

**DOI:** 10.7759/cureus.70686

**Published:** 2024-10-02

**Authors:** Zornitsa Zlatarova, Dariya Chivchibashi-Pavlova, Deyan Dzhenkov

**Affiliations:** 1 Department of Optometry and Occupational Diseases, Faculty of Public Health, University Specialized Eye Hospital, Medical University of Varna, Varna, BGR; 2 Department of Physiology and Pathophysiology, Faculty of Medicine, Medical University of Varna, Varna, BGR; 3 Department of General and Clinical Pathology, Forensic Medicine and Deontology, Medical University of Varna, Varna, BGR

**Keywords:** eyelid tumors, meibomian gland, rare tumors, sebaceoma, sebaceous neoplasms

## Abstract

Sebaceoma is an infrequently diagnosed benign neoplasm with sebaceous differentiation. This tumor usually presents in areas rich in sebaceous glands, such as the face, scalp, or trunk. The periocular tissues are an exceptionally uncommon site for sebaceoma, making sebaceoma of the eyelid an extremely rare entity. To our knowledge, only five cases of sebaceoma of the eyelid have been reported, with only one of these five originating in a Meibomian gland. Due to its rarity and the wide spectrum of skin lesions, sebaceoma is considered a diagnostically challenging lesion. When the diagnosis is uncertain, a biopsy followed by histopathological analysis should be performed to carefully differentiate sebaceoma from other eyelid tumors. Histopathologically, sebaceoma displays a wide range of patterns, from those seen in sebaceous adenoma to features that can be difficult to distinguish from sebaceous gland carcinoma. We report the case of a 64-year-old Caucasian male presenting with sebaceoma of a Meibomian gland of the left lower eyelid margin. The diagnosis of sebaceoma was made based on a routine histopathological examination.

## Introduction

Sebaceoma is a slow-growing, benign neoplasm with sebaceous differentiation [1]. It is a rare tumor entity, with a reported incidence ranging between 0.05% and 0.7% of all skin tumors [2,3]. Clinically, these tumors tend to present as small (5-30 mm), solitary, flesh-colored, or yellowish papules or nodules, but they are sometimes multiple, particularly in Muir-Torre syndrome [2,4,5]. The latter is a rare autosomal dominant disorder, characterized by the presence of multiple skin neoplasms (mainly with sebaceous differentiation) and the presence of primary visceral malignancies [2]. However, benign sebaceous tumors located above the neck are usually not associated with Muir-Torre syndrome [6]. Most patients are in their sixth to ninth decades [4,5], although these tumors may also be seen in younger adults [5]. These tumors typically appear in areas rich in sebaceous glands, such as the face, scalp, or trunk [2,4,7]. Eyelid sebaceoma, per se, is an extremely rare entity [6,7]. To the best of the authors’ knowledge, only five cases of sebaceoma have been reported in the English literature [6-10], and only four of these have been well described [6,7,9,10]. Furthermore, only one case has been reported to have originated in a Meibomian gland [7]. Metastasis and recurrence after treatment have not been reported [4,5].

Due to its rarity and the wide spectrum of skin lesions, sebaceoma is considered a diagnostically challenging tumor [2]. Histopathologically, sebaceoma can exhibit a wide diversity of patterns [5]. Pathomorphological features of sebaceoma range from those observed in sebaceous adenoma to features that may be challenging to differentiate from sebaceous gland carcinoma [6,9]. It is usually a dermal lesion with variable epidermal involvement and rarely affects the subcutaneous fat [4]. Lesions are composed of a mixture of basaloid cells and mature sebocytes, with more than 50% of the tumor made up of basaloid cells [2]. An organized lobular architecture is usually lacking [4]. The basaloid cells are small and uniform with minimal indistinct cytoplasm and round nuclei, occasionally containing small nucleoli [4]. There is no significant pleomorphism, and mitotic activity is infrequent [4,6,10]. The sebocytes appear mature with eosinophilic vacuolated cytoplasm and scalloped nuclei [4].

Sebaceoma should be distinguished from sebaceous adenoma, which typically exhibits a lobular architecture [4,7] and has less than 50% basaloid cells [2,6,9]. Sebaceoma should also be differentiated from sebaceous gland carcinoma, which is characterized by significant nuclear pleomorphism, nucleolar prominence, and conspicuous mitotic activity [4,6]. Additionally, sebaceoma should not be confused with basal cell carcinoma showing sebaceous differentiation, in which peripheral palisading and cleft formation are clear and the sebaceous differentiation is merely an incidental finding [4,6].

This study presents the case of a 64-year-old patient who developed a sebaceoma originating from a Meibomian gland located on the margin of his left lower eyelid. The diagnosis was confirmed through standard histopathological analysis of a biopsy specimen.

## Case presentation

A 64-year-old Caucasian man was admitted to the Surgical Department of the Specialized Eye Hospital in Varna with a solitary nodular lesion on his left lower eyelid. Before his admission, he had been diagnosed with a hordeolum while receiving outpatient care in a different city and underwent unsuccessful treatment with antibiotic and corticosteroid eye drops, specifically Tobramycin and dexamethasone ophthalmic suspension (0.3%/0.1% w/v), which he applied four times daily.

**Figure 1:**
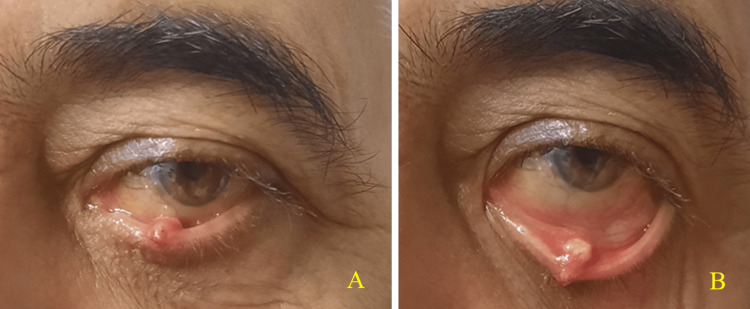
Clinical photographs of the tumor at initial presentation: (A) A nodular lesion located on the inner surface and lid margin of the left lower eyelid. (B) Upon eversion of the lower eyelid, the inner portion of the tumor mass was visible as a white-yellowish nodule.

**Figure 2:**
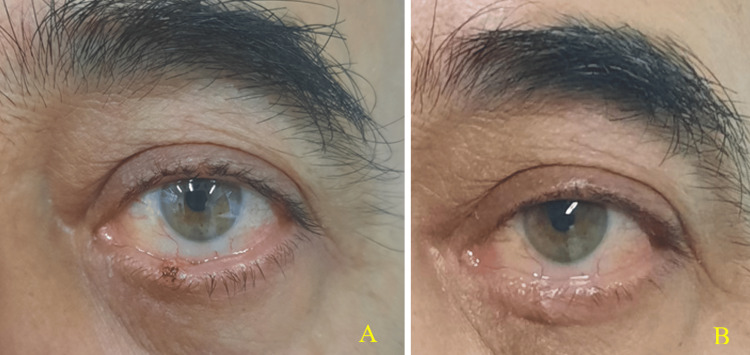
Clinical photographs of the tumor following the excisional biopsy (A) and after six months of postoperative recovery (B).

During the clinical examination, a white-yellowish lesion with a finely granular surface was observed on the inner surface and eyelid margin of the left lower eyelid, measuring approximately 12-13 mm × 5-6 mm (Figure 1). The lesion was perpendicular to the eyelid margin, engaged the tarsal plate, and involved the Meibomian gland. Although it was painless, it caused discomfort during blinking. A diagnosis of a sebaceous tumor was made. Initially, an incisional biopsy was performed, and histopathological examination revealed a sebaceous neoplasm. In the second stage, a full excision of the remaining tumor was performed, leaving a margin of 2-3 mm from the visible edges, encompassing the full thickness of the eyelid (Figure 2, Panel A). The resulting defect was closed directly using standard surgical techniques. Following the incisional biopsy and before the excision of the remaining tumor, the patient was treated with Maxitrol eye drops four times a day in the left eye and Tobradex ointment before sleep.

**Figure 3:**
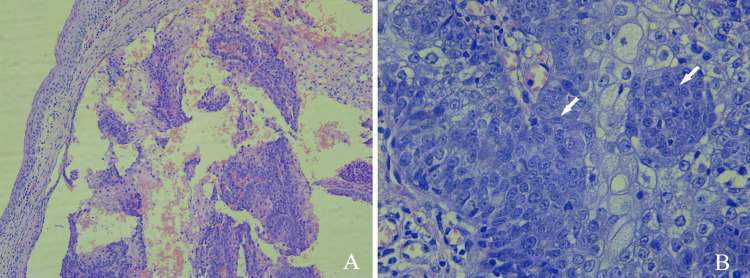
Histopathology microphotographs of permanent paraffin-based sections (hematoxylin and eosin staining, original magnifications ×100 and ×400) showing a well-circumscribed tumor lesion situated within a “cystic” background. The tumor is composed of small blue basaloid cells (white arrows) and paler staining sebaceous cells, the majority of which are vacuolated (A). A higher-power image of the lesion reveals that basaloid cells clearly constitute the predominant cell type, comprising more than 50% of the cellular composition (B).

Histopathological analysis of the excisional biopsy revealed an intradermal cystic lesion composed of papillary proliferation consisting of basaloid cells (>50%), mature sebaceous cells (50%), and fibrovascular stroma (Figure 3). There were no signs of significant cytological atypia or mitotic activity. The tumor extended to the epidermal layer with foci of necrosis. Peripheral to the tumor lesion, a focus of granulomatous reaction was detected with numerous macrophages and areas of lymphoplasmacytic inflammatory infiltrate. Therefore, given the predominance of basaloid cells, which made up more than 50% of the tumor, along with the absence of atypical mitosis and nuclear atypia, a diagnosis of sebaceoma of the eyelid was established.

Postoperatively, for the first 20 days, the patient continued using Maxitrol eye drops four times a day in the left eye and Tobradex ointment before sleep, along with Blepharoclean sterile eye pads with lotion for additional care. For the subsequent 10 days, he continued using Tobradex ointment before sleep and applied Blepharoclean sterile eye pads. The postoperative recovery was complete. After surgical treatment, the lower eyelid of the left eye returned to a normal appearance, position, and function. There were no signs of recurrence during the six-month follow-up period (Figure 2, Panel B).

## Discussion

This case is quite interesting because the rare tumor lesion, sebaceoma, originated from a Meibomian gland and presented on an unusual site, the eyelid, which only adds to its peculiarity.

The term “sebaceoma” was proposed by Troy and Ackerman for a distinctive rare sebaceous tumor entity [5]. Among sebaceous tumors, sebaceoma is one of the most uncommon benign neoplasms. As immunohistochemistry cannot differentiate among various sebaceous tumors, routine histopathological analysis is considered a reliable diagnostic tool for distinguishing them [3,6].

Sebaceoma, when correctly diagnosed, is a tumor of the adnexal epithelium that can extend into the superficial epithelium [4,7,9], as noted in our case. Typically, this tumor has a predilection for the head and neck region, with the eyelid generally being spared [6,7]. Importantly, sebaceoma is characterized by a prominent basaloid component (>50%) compared to mature sebaceous cells [2,6,9].

To our knowledge, only five cases of eyelid sebaceoma have been reported in the English literature to date (Table 1) [6-10]. In these reports, sebaceoma presented as well-defined, small, reddish, or tan-colored nodules involving the lid margin. Occasional mitosis was noted in two cases [6,10], but cytological atypia was not observed in any case. Although mitoses are not a characteristic feature of sebaceoma, sparse mitoses have been documented in the literature [4,6], which may raise suspicion of possible sebaceous gland carcinoma. Nevertheless, the absence of nuclear atypia and necrosis within the tumor argues against sebaceous gland carcinoma [4].

**Table 1:** Details of all reported cases of eyelid sebaceoma in the English literature.

Study	Gender, age (years old)	Location, size	Histopathological findings	Follow-up
Yonekawa et al., 2012 [10]	Male, 53	Upper eyelid margin, size not mentioned	Epidermal layer not involved, occasional mitosis, lack of cellular atypia	Not specified
Mittal et al., 2014 [9]	Male, 81	Lower eyelid margin, 4 mm × 4 mm × 3 mm	Epidermal layer involved, lack of cellular atypia and mitotic activity	24 months, no recurrence
Lee et al., 2016 [8]	Female, 42	Lower eyelid margin, size not specified	Not specified	Not specified
Jakobiec et al., 2020 [7]	Male, 74	Upper eyelid, 13 mm × 9 mm × 6 mm	Epidermal layer involved, cellular atypia and mitotic activity – not mentioned	9 months, no recurrence
Barh et al., 2021 [6]	Male, 59	Upper eyelid sparing margin, 11 mm × 9 mm × 7 mm	Epidermal layer not involved, occasional mitosis, no nuclear atypia	18 months, no recurrence
Present study	Male, 64	Lower eyelid margin, 12 mm × 13 mm × 6 mm	Epidermal layer involved, occasional mitosis, lack of nuclear atypia	6 months, no recurrence

In our patient, the lesion was a comparatively larger nodule, white-yellowish in color with a finely granular surface. The absence of prominent cellular atypia differentiated this tumor from sebaceous gland carcinoma [4,6]. Furthermore, the prominent sebaceous component and the lack of peripheral palisading and cleft formation clearly ruled out basal cell carcinoma with sebaceous differentiation [4]. Regarding sebaceous adenoma as a differential diagnosis, both sebaceoma and sebaceous adenoma share similar clinical features but differ in histopathological findings. Specifically, a less prominent basaloid cell content (<50%) would suggest sebaceous adenoma [2,6,9].

Ultimately, the determination of whether a lesion is benign or malignant cannot be definitively established based solely on clinical assessment. Therefore, the prevailing gold standard for the management of eyelid tumors is surgical excision combined with subsequent histopathological examination. This approach ensures accurate diagnosis and informs appropriate treatment strategies. Furthermore, for follow-up care, patients should undergo monitoring every three months during the first year, every six months during the second and third years, and annually thereafter, provided there are no indications of recurrence. It is essential to educate patients that if they observe any new lesions, they should seek an earlier consultation than previously scheduled. This follow-up protocol is typically employed for malignant cases but may also be applicable in this context to ensure comprehensive patient management.

## Conclusions

Sebaceoma of the eyelid is an extremely rare tumor entity that presents a diagnostic challenge. To avoid misdiagnosis, a biopsy of the lesion should be performed, carefully distinguishing it histopathologically from other sebaceous neoplasms such as sebaceous adenoma, sebaceous gland carcinoma, or even skin malignancies such as basal cell carcinoma with sebaceous differentiation.
